# Supplementary material for: The partition representation of enzymatic reaction networks and its application for searching bi-stable reaction systems
Source: PLoS One. 2022 Jan 26;17(1):e0263111. doi: 10.1371/journal.pone.0263111 (PMC8791506; doi:10.1371/journal.pone.0263111)
Supplement: Attachment — Submitted filename: Response to Reviewers.docx [file pone.0263111.s007.docx]

Re: Letter to Editors

June 25, 2021

PLOS ONE

Editorial Office

Dear Editors:

Please find enclosed a revised manuscript entitled “The partition representation of enzymatic reaction networks and its application for searching bi-stable reaction systems” [PONE-D-21-10250] - [EMID:a87fa0fe8b02dd7a], for publication in PLOS ONE.

I revised the manuscript by taking account of the reviewer’s relevant comments. I tried to revise the manuscript with all my efforts and would like to give a point-by-point response to comments below. The reviewer’s comments are written in italics.

**Answers to the comments by Reviewer #1:**

*The paper is not intelligible the way it is currently written. It is difficult to say whether the findings merit publication if written better, because in the current form it is difficult to understand even what the main point is. The author says "proposed search algorithm worked well" in the abstract without specifying the basis for this statement. Similarly, there is a vague and general remark at the end of the abstract "seems to be applicable to the search for dynamic properties".*

The paper has been reviewed in general and additions have been made where necessary.

*But mostly the main text of the paper is extremely unclear. Maybe the author can summarize the goal of the paper in a short paragraph, and explain one example clearly. Unfortunately, the way it is currently written, I am afraid the paper may be of little use to anyone who is not completely well-versed with the author's methods already.*

The paper has been reviewed in general and additions have been made where necessary.

**Answers to the comments by Reviewer #2:**

*The concept of partitioning isn’t really clear. It isn’t thoroughly explained in the text, nor any references were provided. I searched this topic of partitioning enzymatic networks and found nothing. I assume this is the key novelty of the paper. So this piece really requires some additional work to make sure the concept is clear to the reader.*

The paper has been reviewed in general and additions have been made where necessary.

*What is the point of Figure 1? Are these building blocks of partitioning? Is this a complete set of all building blocks?*

The building block of partitions is only the post-translational modification reactions shown in Eq (1), and the N post-translational modification reactions are interconnected by species identification to form an enzymatic reaction network. The family of the set of chemical species to be identified is the partition representation. Fig 1 shows an example of the case where N=1 and N=2. In other words, Fig 1 is an example of an enzymatic reaction network composed of rather than building blocks.

To make the above clear, I added an example in Fig 1 for the case of N=1, and in the text, I added and modified the partition representations corresponding to each enzymatic reaction network. I also added and corrected the explanation of the network expression in the figure, which I think was missing.

*Figure 2 also leaves a lot of questions. How does Fig 2a networks leads to {{P1,S1},{P1,S2,E1,E4 },{P4,S3},{P3,S4,E3,E2}} partitioning? Same pertains to even a more complicated Figure 2b. Are partitionings unique?*

*I’d suggest perhaps coming up with a figure that shows in details how the network is constructed from partitions.*

As in Fig 1, the set M, which is the source of the partition, is explicitly shown for Fig 2. There is a one-to-one correspondence between the partition and the enzymatic reaction network that is constructed.

*Is the terms “partition” the right one? Usually this means that something was divided into smaller non-overlapping parts. I think the key concept of the paper is construction of the networks from smaller blocks. This slightly non-conventional use of terminology could be confusing for the reader.*

In this paper, partition is also used in the sense of a family of subsets, where the sets are disjoint to each other and their union set is the original set. In other words, it is a division into small non-overlapping parts. This is the representation of an enzymatic reaction network.

*Introduction of the distance in the partition space (equation 3) would also benefit from more explanations. I would suggest using a small example and show how the equation (3) can be applied.*

Concrete examples were added to the explanation of the distance between divisions (Eq 3). In addition, diagrams of the enzymatic reaction networks corresponding to the examples were added as Fig 3.

*Evaluation of bistability described in Figure 3 isn’t clear either.*

The method of evaluating bistability using the function in Fig 3 (Fig 5 in the revised version) has been substantially added and revised.

*I wonder about the term “controllable bistability”. The author gives the definition and refers to the citation 20. Frankly, this is the first time I see the terms controllable bistability. Is there an uncontrollable one in the chemical reaction networks? Please provide an example of uncontrollable bistability so the use of “controllable” part is justified.*

In Reference 20, it is called “resettable bistability”. Thus, I will change to that term as well. Futhermore, I have added Fig 4 and a description to explain the relationship between bistability and resettability.

*Results section, step 4 of the algorithm is the key for evaluating the bistability of the constructed network. What is the “degree” of controllable bistability? Then algorithm itself is kind of brief. Evaluation for bistability of a parameter-free network is a huge problem. I don’t fully understand what compromises have been taken in step 4, so the evaluation is quite short. It feels like it was a direct simulation of ODEs to reach the equilibrium and then concentration of output species plotted against the input. If this is the case, then it is a very risky approach. How to be sure that the hyperparameter space was searched exhaustively enough?*

I have added an explanation to the method of evaluating controllable (resettable) bistability in step 4 of the algorithm.

Also, as you pointed out, as the stable equilibrium point (steady state) of the system, I used the value at which the time evolution of the system reached equilibrium in the direct simulation of the ODE. This is because the system is nonlinear and has a high order, so solving the algebraic equations corresponding to the steady state would eventually have to be done numerically, and determining whether the system is stable or unstable would require calculations such as finding the eigenvalues of the Jacobi matrix, which I thought would not be very efficient. I will add an organized explanation of the parameters and hyperparameters of the system in the text.

*As I mentioned before, I’d love to see this published and used by the community. However, if the paper is published as is, it will be lost because it is hard to follow. If I may suggest an example of a paper that is written like an easy to follow tutorial is “Chemical Reaction Network Theory elucidates sources of multistability in interferon signaling” by Irene Otero-Muras et al, PLOS Comp Biol.*

<https://journals.plos.org/ploscompbiol/article?id=10.1371/journal.pcbi.1005454&rev=2>

Thank you for introducing the appropriate literature. I will refer to it.

We hope the revised paper is of interest for the readers of PLOS ONE. I am looking forward to hearing from you again.

Yours sincerely,

Takashi NAKA
