# Supplementary material for: The partition representation of enzymatic reaction networks and its application for searching bi-stable reaction systems
Source: PLoS One. 2022 Jan 26;17(1):e0263111. doi: 10.1371/journal.pone.0263111 (PMC8791506; doi:10.1371/journal.pone.0263111)
Supplement: Attachment — Submitted filename: Response to Reviewers.docx [file pone.0263111.s008.docx]

Re: Letter to Editors

October 18, 2021

PLOS ONE

Editorial Office

Dear Editors:

Please find enclosed a revised manuscript entitled “The partition representation of enzymatic reaction networks and its application for searching bi-stable reaction systems” [PONE-D-21-10250R1] - [EMID d593f28512f8cd09], for publication in PLOS ONE.

I revised the manuscript by taking account of the reviewer’s relevant comments. I tried to revise the manuscript with all my efforts and would like to give a point-by-point response to comments below. The reviewer’s comments are written in italics.

**Answers to the comments by Reviewer #2:**

*While the author has addressed a lot of concerns, one of my major ones remains to be fully addressed. Specifically, I don’t understand how the evaluation function for resettable bistability (section starting on page 17, line 379) works. Either I am missing some details or this evaluation must be extremely computationally intensive.*

*First of all, my understanding is that the author decided to take a straightforward approach by evaluating the bistability by numerically solving the generated differential equations. Let’s say even in a fairly moderate biologically-relevant ODE system, in total there are about 10 total parameters (starting material amounts and kinetic constants). It seems like the author proposed to use 21 values per dimension. This translates into 16 trillion times solving the ODE system! Even if the number of dimensions is 5 (that limits the method to really primitive systems), it requires 4 million solutions. Let’s say one evaluation takes 1 second, this translates into about 1 month of computation time. So the straightforward numerical evaluation of bistability must be cursed by high dimensionality.*

*Again, for the sake of the benefit of the doubt, I may be missing something in the algorithm and/or the assumptions. I’d note that evaluation for resettable or not bistability is a hard problem. There are a variety of known approaches of how to evaluate bistability. Each of those approaches has its limitations. But no one in the field even considers evaluation of bistability for direct simulation of ODEs for the reasons outlined above.*

*The entire “Evaluation function for resettable bistability” needs to be improved.*

The method of constructing the evaluation function for resettable bistability has been modified to address each of the points pointed out below, and the overall description has also been modified to make it easier to understand.

1. *Author needs to address the dimensionality of the problem.*

The reviewer estimates that even if there are 5 parameters, if each value is 21 ways, we get 5^21 ≈ 4000000, which is equivalent to an exhaustive evaluation on the parameter values. I used to do this kind of exhaustive analysis, but as the reviewer pointed out, it is computationally explosive, so this is not the method used in this study. In this study, only five random combinations of parameter values are used for a single enzymatic reaction network, and the evaluation is done with those values. This value of 5 is the value of the parameter λ.

It should be pointed out, however, that the same enzymatic reaction network may be revisited, as we do not try to remove enzymatic reaction networks that have been evaluated once from the evaluation. Even in this case, the search depth σ of the two-branch search with two different partition modifiers is set to 4, so the maximum number of nodes in the binary tree is 62=2^1+2^2+2^3+2^4+2^5, and the maximum number of iterations of the main loop to change the partition is 150, so the computational cost is at most 9600=62*150 times. In addition, the actual number of parameters such as reaction rate constants and total concentration is not five, but four post-translational modification reactions, so the maximum number of reaction rate constants is 12=3*4 and the maximum number of total concentrations is 8=2*4, so the total number is a maximum of 20. This means that the cost of evaluation in the parameter space of one enzymatic reaction network is 5*9600 versus 21^20.

Why such a sparse sampling of parameter values can successfully reveal an enzymatic reaction network with the desired properties may require some explanation. Based on the exhaustive analysis I have done, one explanation is that the bistable nature of enzymatic reaction networks is dominated by the network structure and is quite robust in terms of changes in parameter values. This means that the bistable property is maintained over a quite wide range of parameter values. Alternatively, we can consider that a robust enzymatic reaction network is selectively found. However, it should be noted that it is not robust with respect to input chemical species, as we have placed the condition that it is resettable.

2. *There needs to be some discussion of kinetic constants along the species concentrations.*

The rate constants of the post-translational modification reactions and the total concentration of enzymes in the enzymatic reaction network are set to include approximately the values reported for the kinases that make up the MAPK cascade, which is known as a typical signaling system. This description was added in the revised text.

3. *What is theta in step 2 (line 394)? How to estimate this theta?*

The total concentration of each enzyme is randomly assigned as the initial value to satisfy the conservation law for each enzyme, and the process of finding 21 pairs of steady state values for the output chemical species is repeated *theta* times. As a result, we obtain *theta* steady states when the reaction rate constants of the constituent post-translational modification reactions and the total concentration of the enzyme are fixed. When all these values are the same, it means monostability, and when they are divided into two kinds of values, it means bistability. This explanation has been added to the relevant section. The value is set to 5 as appropriate, but as described in the discussion in the text, we tried other values and found no significant difference in the search results.

4. *Why does the author need to take quadruple of the variance? Why quadruple? What is the purpose of the entire step?*

From the *theta* steady state values obtained up to the previous step, the variance of those values can be used to quantitatively evaluate the degree of bistability. Since the steady state values are normalized in the range of 0 to 1, the variance takes a maximum value of 1/4 in a perfect bistable state, i.e., when half of the values are 0 and the rest are 1, and a minimum value of 0 in a monostable state, i.e., when all values are the same. So we multiply this value by 4 for the purpose of normalizing it. This explanation has been added to the corresponding step.

5. *Since that point I’ve lost track.*

As I answered in the beginning, I tried to revise the description to make it clearer for the other parts you pointed out.

*Also, to make sure that the manuscript complies with "Have the authors made all data underlying the findings in their manuscript fully available?” question, I’d suggest making all the code and scripts available. I understand that the manuscript is mostly theoretical. However, if the description of the algorithms is not clear, the code may help.*

For reference, the program code is submitted as supplementary material.

**Answers to the comments by Reviewer #3:**

*This manuscript introduces a novel approach to explore dynamical properties of an enzymatic network. The novelty comes from the representation of an enzymatic network as a partition of the set instead of a regulatory matrix. This representation offers a possibility for an effective search of various dynamical properties of a network. The results demonstrate the exploration of reaction networks for the properties such as bistability and resettability.*

*The paper demonstrates a novel approach and important results for the exploration of bistable biological systems. However, the way this paper is written is not yet suitable for publication. “Introduction” and “Materials and Methods” sections were not comprehensible to me when reading the paper for the first time. The “Results” section clarified some confusion that appeared while reading the first part of the paper. Below are comments that might improve the readability of the paper and questions that appeared while reading it:*

1. *Resettability and bistability are rather central concepts in this paper. These terms appear a lot in all parts of the paper except in “Materials and Methods”. It seems like resettability and bistability drop out of that section and appear again in “Results”, making it harder for the reader to reconnect to the main story of the paper. I would recommend introducing these terms already in “Materials and Methods” next to enzymatic reaction networks which exhibit these properties. Figure 4a next to a visualization of an enzymatic reaction network which has this property would serve as a good illustrative example of what you are looking for. Then, the partition representation can be introduced for the purpose of finding networks with these properties.*

   Following your suggestion, I moved "Relationship between Bistability and Resettability" to " Materials and Methods" and changed it to "Resettable Bistability". I also referred to the enzymatic reaction network of Fig 2a as the example.
2. *In the subsection “Relationship between bistability and resettability” it is not clear which enzymatic network is discussed. “The relationship between bistability and resettability when the input chemical species is E1 and the output chemical species is P2 is shown in Fig 4” ? it is not clear which network structure is discussed in this sentence and what are E1 and P2. Is it the network from Figures 1,2,3 or 6?*

   In "Resettable Bistability", which was moved and renamed in response to comment 1, I added an explanation referring to the enzymatic reaction network of Fig 2a as the example, and also added an explanation for E1 and P2 in that part.
3. *The algorithm descriptions in the section “Exploration of enzymatic reaction networks in the partition representation space” can be improved by either representing them as block diagrams or writing pseudo-code.*

   I tried to break down each step of the algorithm and modify it to be at the level of pseudocode.
4. *Section “Exploration of enzymatic networks in the partition representation space” can be framed with some intro paragraph explaining what to expect from the algorithm and some concluding paragraph summarizing the subsection. The section ends too abruptly with the steps of the algorithm. Moreover, it would create better connection with the rest of the paper if you already briefly mention how the search for resettability and bistability can be incorporated in this scheme.*

   Following your suggestion, I rearranged the description of the entire section and added the intro and final paragraphs.
5. *Lines 51-53: “There, the control relationship between the cyclic reaction systems is represented by a control matrix whose elements are the cyclic reaction systems that make up the system.” ? please rephrase. “Systems that make up a system” sounds confusing.*

   I deleted the unnecessary part of the sentence you mentioned.
6. *In the sentence in lines 75-78 you list various properties and only biochemical adaptation has a definition next to it. Please, make this consistent. You could introduce the definitions to all properties in a separate sentence, or even omit the definition and add a good reference for the interested reader.*

   I added explanations and references for all five properties mentioned.
7. *Sentence in lines 79-82 has too many nested clauses, which makes it hard to grasp for the reader. Please, consider rephrasing it.*

   I broke the sentence you pointed out into two sentences to eliminate nesting, and also modified the next sentence slightly.
8. *In the end of Introduction, term “resettability” appears rather unexpectedly. Moreover, ending the section with a definition worsens the readability of an article. Try to properly incorporate this term in the Introduction.*

   Resettability was introduced as an additional property of bistability, and an explanation was added.
9. *Line 93: please, rephrase the second half of the sentence and avoid “let’s think”.*

   I paraphrased using "Considering"*.*
10. *Figures 1,2,3,6: rectangles for S and P would look better if they were white inside rather than transparent.*

    I followed your suggestion and whitened the inside of the rectangle.
11. *From line 124 till line 148 almost every other sentence starts with “In general”. Please, remove that or come up with another opening word.*

    I’ve removed all "in general."
12. *Line 148: “dimer formation-separation reaction”*

    I have corrected it as you suggested.
13. *Line 163: “Fig 2 shows examples of more complex enzymatic reaction networks and its parftition representations” ? typo in “partition”, and I believe it should be “reaction networks and their partition representations”.*

    You are correct. I’ve corrected it.
14. *Line 170: remove extra “the”.*

    I’ve removed the extra “the”.
15. *Line 175-176: what do you mean by inactive and active MAPKKs? Maybe add some context for a reader who is not familiar with this system.*

    I followed your advice and added a brief description of the MAPK cascade.
16. *Line 185: “The advantage of the partition representation is that every partition is a representation…” ? please, rephrase.*

    I paraphrased that sentence.
17. *Line 197: “…the set M shown in Eq” -> “…the set M as shown in Eq”*

    I’ve fixed the sentence.
18. *Sentence in line 234: please expand or rephrase for better readability. The introduction of sk is a bit confusing.*

    I rewrote the text, including the introduction of sk*.*
19. *Line 270: rk -> rk*

    I’ve corrected the part you pointed out.
20. *Line 301: “…φ(P,ν) is a function of the partition P, with the parameter ν a pair of reaction…” ? I believe you meant “… the parameter ν, which is a pair of reaction …”*

    You are correct. I’ve corrected the sentence.
21. *Line 309: “and takes a maximum value among them as a value” -> “and takes a maximum among them as a value”. Line 309-310 ? please, rephrase and reduce the number of words “value”.*

    I revised the sentences and reduced the number of the word "value".
22. *Sentence in lines 317-320 is too long and might confuse the reader. If I understood it correctly, it can be split into 2 sentences: “The partition search function ψ(P0, P, σ ) described below is a recursive search function whose arguments are the initial partition P0, the current partition P, and the search depth σ. ψ(P0, P, σ ) returns the partition whose evaluation function value is greater than or equal to Ф(P0).”*

    As you suggested, I split the sentence into two.
23. *Line 325: “is greater than of P0 ”*

    I’ve fixed the sentence.
24. *Line 330: the last step says “return P0 as a value” without any condition, which sounds confusing. Please add a condition, smth like “if all steps above are completed…” or “if none of the conditions above are satisfied…”*

    I have added the condition as you suggested.
25. *Line 336: “Resettablility is the property”. Resettable is an adjective.*

    I’ve corrected the part you pointed out.
26. *Lines 371-372: “This is an example of a bistable that is not resettable” -> “This is an example of a bistable system (or a bistability?) that is not resettable”*

    I’ve corrected the part you pointed out.
27. *The sentence in lines 508-513 is too long. Please, rephrase for better readability.*

    I’ve corrected the part you pointed out to make it easier to read.
28. *Figure 7 ? why do some dots have different colors than others?*

    The colors correspond to different initial values for obtaining the steady state values. However, there is no point in showing it in this figure, so we changed it to the same color*.*
29. *Figure 8 ? as I understood, some arrows (like (a) and (g), and (b) and (h)) overlap significantly. Could you come up with a better representation? Maybe highlight it in the description of the figure or use dashed lines.*

    As you pointed out, some of the arrows overlap in Fig 8a. In particular, the evaluation value reaches 0.6 in all cases up to the first two steps, and almost everything else overlaps up to that point. So I changed the range of the vertical axis to 0.55 or higher, and modified it to reduce the overlap as much as possible after that. I added a note about this correction in the text and in the explanation.
30. *Lines 558-563 seem like an outlook. Consider first summarizing your findings and writing an outlook in the end of the Discussion. Also, line 563: I -> We, please, be consistent.*

    Following your suggestion, I have revised the order to summarize the results first, and then the outlook at the end. The subject in line 563 has also been changed to "We".
31. *Line 569-570: “Good results” ? please, avoid using this in a scientific paper.*

    I’ ve corrected the description to be more specific.

We hope the revised paper is of interest for the readers of PLOS ONE. I am looking forward to hearing from you again.

Yours sincerely,

Takashi NAKA
